# Influence of Prolonged Serotonin and Ergovaline Pre-Exposure on Vasoconstriction Ex Vivo †

**DOI:** 10.3390/toxins14010009

**Published:** 2021-12-23

**Authors:** Eriton E. L. Valente, David L. Harmon, James L. Klotz

**Affiliations:** 1 Animal Science Department, State University of Western Parana, Marechal Cândido Rondon 85960-000, PR, Brazil; eriton.valente@unioeste.br; 2 Department of Animal and Food Science, University of Kentucky, Lexington, KY 40546, USA; dharmon@uky.edu; 3 Forage-Animal Production Research Unit, USDA-ARS, Lexington, KY 40546, USA

**Keywords:** blood vessel, ergovaline, myograph, serotonin

## Abstract

Ergot alkaloid mycotoxins interfere in many functions associated with serotonergic neurotransmitters. Therefore, the objective was to evaluate whether the association of serotonin (5-hydroxytryptamine, 5-HT) and ergot alkaloids during a 24 h pre-incubation could affect the vascular contractile response to ergot alkaloids. To evaluate the effects of 24 h exposure to 5-HT and ergot alkaloids (ergovaline, ERV), two assays were conducted. The first assay determined the half-maximal inhibitory concentration (IC_50_) following the 24 h pre-exposure period, while the second assay evaluated the effect of IC_50_ concentrations of 5-HT and ERV either individually or in combination. There was an interaction between previous exposure to 5-HT and ERV. Previous exposure to 5-HT at the IC_50_ concentration of 7.57 × 10^−7^ M reduced the contractile response by more than 50% of control, while the exposure to ERV at IC_50_ dose of 1.57 × 10^−10^ M tended to decrease (*p* = 0.081) vessel contractility with a response higher than 50% of control. The 24 h previous exposure to both 5-HT and ERV did not potentiate the inhibitory response of blood vessels in comparison with incubation with each compound alone. These results suggest receptor competition between 5-HT and ERV. More studies are necessary to determine the potential of 5-HT to treat toxicosis caused by ergot alkaloids.

## 1. Introduction

Ergot alkaloid mycotoxins have a significant impact on livestock health and productivity globally [1]. The similarities between the tetracyclic ergoline ring, common to naturally occurring ergot alkaloids, and the ring structure of the biogenic amine neurotransmitters allows ergot alkaloids, like ergovaline to interfere in the many functions associated with these neurotransmitters [2]. After a period of feeding ergot alkaloids, some serotonergic receptors become less responsive to serotonin (5-HT) [3,4,5]. Drugs that are derivatives of ergot alkaloids have an association and dissociation rate to serotonin 5-HT_2_ receptors that is much slower than 5-HT [6], causing long-term stimulation. This persistent stimulation of serotonergic receptors has been shown to result in blunted signaling [7] and this alters how tissues respond to receptor-driven stimuli. This is exemplified in ergot alkaloid mycotoxicosis by a sustained vasoconstriction that limits effective blood flow to visceral and peripheral tissues [2]. Many symptoms of ergotism, like fescue toxicosis, are related to vasoconstriction, which is associated with lower blood flow [8,9] causing gangrene [10] and affecting thermal regulation [11]. Currently, there is no effective treatment for mycotoxicosis caused by ergot alkaloids in livestock.

Agonist therapy is the use of stimulant-like medications to treat stimulant addictions and has proven an effective treatment for dependence on neuromodulator drugs, such as nicotine and opioids [12]. This strategy involves the administration of medications that share neurobiological mechanisms with the undesirable drug in pursuit of neurochemical normalization [13]. The exposure to ergot alkaloids can produce neurochemical deficits in 5-HT [14]. Thus, the ideal agonist therapy would normalize 5-HT dysfunction.

Traditionally, agonist therapy uses agonists with lower potency [12]. The less persistent receptor association with 5-HT in comparison with ergot alkaloid derivatives [6] qualifies 5-HT as a potential molecule for agonist therapy for ergot alkaloid toxicosis. Hypothetically, the increase of 5-HT in the synaptic cleft could intensify competition with ergot alkaloids at the serotonergic receptors and reduce the overstimulation through a reduction in ergot alkaloid receptor association. Prolonged overstimulation caused by elevated 5-HT is much more difficult to achieve in comparison to ergot alkaloids because of the many mechanisms that control 5-HT neuron firing [7]. However, there is no evidence to show if a controlled increase in 5-HT can compete with ergot alkaloids for receptors to offset and reduce the overstimulation caused by ergot alkaloids. The potential uses of 5-HT as agonist therapy for ergotism in cattle have not been evaluated previously. Therefore, the objective was to evaluate whether the association of 5-HT and ergovaline in a 24 h pre-incubation could affect the vascular contractile response.

## 2. Results

### 2.1. Pre-Exposure to Serotonin

**Figure 1:**
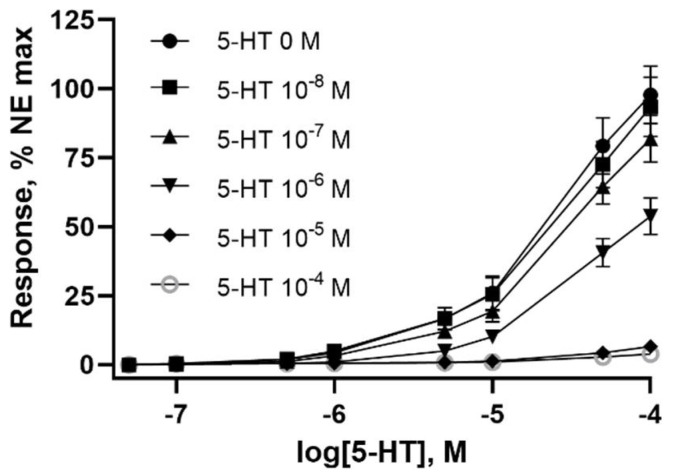
Effects of pre-incubation with serotonin (5-HT) at concentrations of 0.1 × 10^−8^, 1 × 10^−7^, 1 × 10^−6^, 1 × 10^−5^, 1 × 10^−4^ M on contractile responses in the isolated lateral saphenous vein from cattle exposed to increasing concentrations of 5-HT. Points represent the mean values and vertical bars show the SEM, *n* = 12.

**Figure 2:**
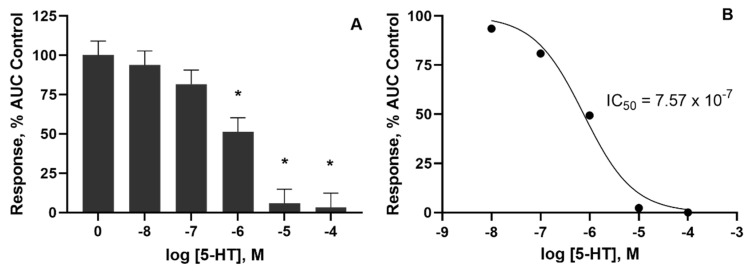
(**A**) Effect of pre-incubation with serotonin (5-HT) on contractile concentration–response curve represented as area under curve (AUC) relative to control. * Indicates a difference relative to control (*p* < 0.05). (**B**)The half-maximal inhibitory concentration (IC_50_) was 7.57 × 10^−7^ M, *n* = 12.

Bovine lateral saphenous veins were constricted as evidenced by decreased (*p* < 0.05) internal and external diameters after the 24 h pre-incubation with 5-HT at doses of 1 × 10^−7^ M or higher (Table 1). However, no difference (*p* > 0.05) was observed in vessel wall thickness. The pre-incubation with 5-HT produced a dose-dependent decrease in vessel contractility when exposed to increasing concentrations of 5-HT (Figure 1). Pre-incubation concentrations of 1 × 10^−6^ M 5-HT and higher resulted in contractile responses to 5-HT that were lower (*p* < 0.05) than control (Figure 2A). The previous exposure dose of 1 × 10^−4^ and 1 × 10^−5^ M 5-HT almost completely suppressed the contractile response of the vessel. The resultant IC_50_ determined from a 24 h previous exposure to increasing concentrations of 5-HT was 7.57 × 10^−7^ M (Figure 2B).

### 2.2. Pre-Exposure to Ergovaline

**Figure 3:**
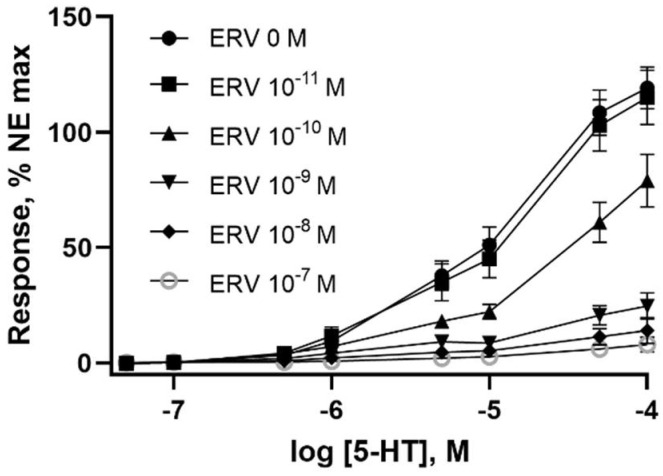
Effects of pre-incubation with ergovaline (ERV) at concentrations of 0, 1 × 10^−11^, 1 × 10^−10^, 1 × 10^−9^, 1 × 10^−8^, 1 × 10^−7^ M on the contractile responses to serotonin (5-HT) in the isolated lateral saphenous vein from cattle. Points represent the mean values and vertical bars show the SEM, *n* = 12.

**Figure 4:**
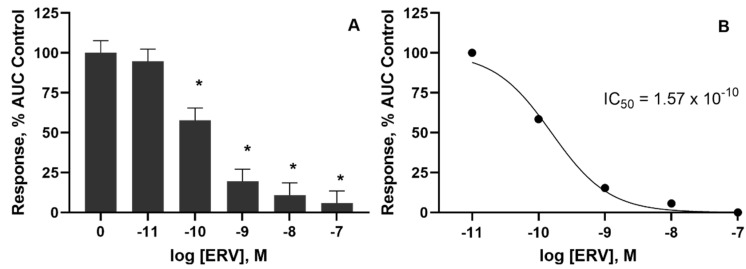
(**A**) Effect of pre-incubation with ergovaline on contractile concentration–response curve represented as area under curve (AUC) relative to control. * Indicates a difference relative to control *p* < 0.05. (**B**) The half-maximal inhibitory concentration (IC_50_) was 1.57 × 10^−10^ M, *n* = 12.

Bovine lateral saphenous veins that were pre-incubated with ERV (in a tall fescue seed extract) at doses of 1 × 10^−9^ M or higher decreased (*p* < 0.05) the internal diameter while doses of 1 × 10^−10^ M or higher decreased the external diameter of the vessels (Table 2). Similar to 5-HT, the vessel wall thickness was not affected (*p* > 0.05) by a 24 h pre-incubation with ERV. Like pre-incubation with 5-HT, the pre-incubation with ERV produced a dose-dependent decrease in contractility in vessels incubated with increasing concentrations of ERV (Figure 3). Only the pre-incubation with 1 × 10^−11^ M ERV produced little effect on vessel contractility, whereas the 1 × 10^−7^ M pre-incubation dose almost completely suppressed the contraction of the vessel (Figure 4A). The IC_50_ for ERV from a 24 h exposure was 1.5 × 10^−10^ M (Figure 4B).

### 2.3. Pre-Exposure to Combined Serotonin and Ergovaline

**Figure 5:**
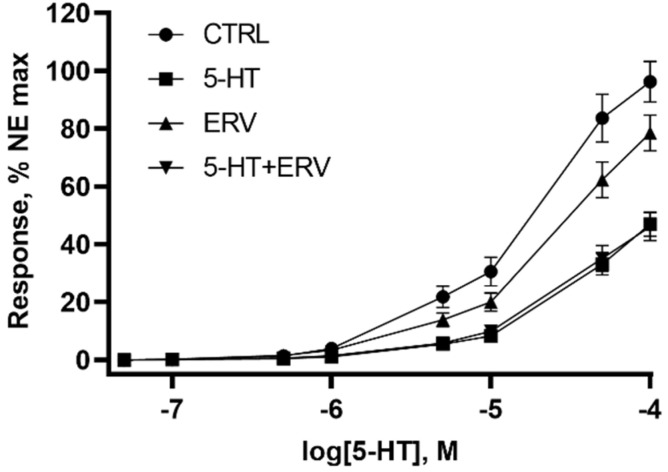
Effect of pre-incubation with buffer (CTRL), serotonin (5-HT), ergovaline (ERV), and 5-HT + ERV on contractile concentration–response curve to 5-HT in the isolated lateral saphenous vein from cattle, *n* = 13 to 15.

The final assay evaluated the effects of the IC_50_ concentrations of ERV and 5-HT alone and 5-HT + ERV in combination during a pre-incubation on vessel dimensions (Table 3) and subsequent contractile response to increasing concentrations of 5-HT (Figure 5). There were no interactions (*p* > 0.05) between treatments for blood vessel length, internal diameter, or external diameter. Bovine lateral saphenous veins pre-incubated with 5-HT at the IC_50_ concentration had greater (*p* < 0.05) length, lower (*p* < 0.05) internal and external diameters, and no difference (*p* > 0.05) in wall thickness. Conversely, those pre-incubated with ERV at the IC_50_ concentration tended to have a shorter (*p* = 0.081) length with no difference (*p* > 0.05) in internal and external diameter, or wall thickness.

There was an interaction (*p* < 0.05) between pre-incubation with 5-HT and ERV on the contractile response to increasing 5-HT, as seen in the AUC response. Previous exposure to 5-HT or 5-HT + ERV reduced (*p* < 0.05) the contractile response by an average of 60% while the pre-incubation with ERV at the IC50 concentration decreased the AUC by only 26%. Interestingly, the 24 h pre-incubation with both 5-HT (7.57 × 10^−7^ M) and ERV (1.57 × 10^−10^ M) did not potentiate the inhibitory response (Figure 5). There was no interaction (*p* > 0.05) between 5-HT and ERV on the maximum response to norepinephrine (Table 3). The pre-incubation with 5-HT decreased (*p* < 0.05) the blood vessel contractility response to norepinephrine. Conversely, the pre-incubation with ERV did not affect (*p* > 0.05) the response to norepinephrine.

## 3. Discussion

This study aimed to perform the first evaluation of the use of 5-HT to mitigate the negative effects of ergot alkaloids on blood vessel contractility following a prolonged ex vivo pre-incubation. The pre-incubation with 5-HT modified the contractility of vessels exposed at physiological concentrations. The results suggest that 5-HT might compete with ergot alkaloids for serotonergic receptor binding.

The 24 h incubation with 5-HT and ERV represented normal cellular conditions in an animal. It has been established that 5-HT receptors mediate the contraction of the bovine lateral saphenous vein [15,16]. The reduction of vessel diameter occurred at 5-HT doses below physiological concentrations (1 × 10^−7^ M). Previous studies have found that bovine plasma 5-HT concentrations are close to 1 × 10^−6^ M [17,18]. Platelets contain a highly efficient transporter that controls 5-HT in the plasma, which represents the free active 5-HT [19]. The prolonged exposure of a static 5-HT concentration during the pre-incubation may have caused an overstimulation of the receptors causing a persistent contracted state.

The reduction of vessel contractility in the myograph is evidence that prolonged overstimulation can decrease the serotonergic receptor response. The concentration of 5-HT near the nerve terminal may substantially alter the activation or desensitization of serotonergic receptors [20]. Chronic infusion of 5-HT produces a residual desensitization of the receptors in many organs [21]. During the contractility evaluation in the myograph, the response to high doses of 5-HT applied for 15 min were rapidly washed out. However, the effect of prolonged exposure to 5-HT during the 24 h pre-incubation was not as readily washed out and a drastic reduction of vessel vasoactivity to 5-HT was observed.

Similar to 5-HT, pre-incubation with ERV provoked an intense reduction of the lateral saphenous vein diameter. In contrast, Trotta et al. [5] did not find a difference in the diameter of bovine ruminal or mesenteric blood vessels after a 2 h incubation with 1 × 10^−8^ or 1 × 10^−6^ M ERV. However, in the current study, the pre-incubation with ERV with lateral saphenous veins decreased the vessel diameter and the contractility at much lower concentrations. The inclusion of a control treatment ensured that the changes in contractility were caused by the ERV and not by loss of viability. The dose-dependent decrease in contractility after pre-incubation with ERV and the occurrence of this effect at a very low dose (1 × 10^−10^ M) was evidence that the effect of ergot alkaloids on blood vessels is mediated by both the dose and the time that the receptors are exposed. Without previous exposure, the ergopeptine ergot alkaloids normally reduce the contractility of vessels at concentrations higher than 1 × 10^−7^ to 1 × 10^−6^ M [4,22,23], which are considerably higher than the ergovaline levels expected in plasma of ruminants consuming ergot alkaloids [24]. The difference in the concentration of ergot alkaloids and the observed responses in the literature indicates that the duration of exposure can influence the ergot alkaloid effect.

Reductions in the contractile response to 5-HT in lateral saphenous veins collected from endophyte-infected tall fescue of grazing steers has been reported [25]. The reduction in the contractile response to 5-HT of vessels can be associated with a reduction of mRNA of both 5-HT receptors [26,27] and calcium channels [26]. The persistent contracted state of the vessels after ergot alkaloid administration and resistance to washout [15] can be caused by the lower receptor dissociation of the ergot alkaloids [6]. However, prolonged exposure to 5-HT also decreased the relaxation of vessels and the contractility in the organ bath, demonstrating that many mechanisms are involved in changing vessel contractility, rather than receptor kinetics alone.

The lower contractility of blood vessels exposed to ergot alkaloids can be partly explained by the reduction of 5-HT receptors [6]. However, the reduction of contractility after chronic exposure to 5-HT, which is easily washed out compared to ergovaline, indicates other mechanisms also act to modify the contractile response. One plausible mechanism is that the overstimulation of serotonergic receptors, either by chronic 5-HT or ergot alkaloid exposure, can alter calcium dynamics and consequently the smooth muscle contractility.

The administration of ergot alkaloids does not affect the maximum contractile response of vessels to KCl [4,5]. The KCl-derived stimulation bypasses G protein-coupled receptors and activates smooth muscle by the activation of voltage-operated Ca^2+^ channels [28]. Although it is not clear how ergot alkaloids act on smooth muscle to influence the contractile response, it is possible that they act on G protein-coupled receptors and in different parts of the calcium pathway, other than by voltage-operated Ca^2+^ channels.

Although prolonged 5-HT exposure mimicked the response of ergot alkaloids, the difference in concentration for a similar response was large. The IC_50_ concentration for ERV was almost 5000-fold lower than for 5-HT. The chronic infusion of 5-HT produces a residual desensitization of serotonergic receptors in smooth muscle [21]. The prolonged stimulation of 5-HT receptors leads to phosphorylation, which modifies cell surface expression, coupling profiles, and interactions with protein partners, usually leading to blunted signaling [7]. Serotonin has many mechanisms to control synaptic signaling and prevent neural malfunction. Conversely, the ergot derivatives do not have these mechanisms to control receptor stimuli and their long durations of action are not easily attributed to circulating levels [6]. The vasoconstriction caused by ergot alkaloids can provoke several modifications in cattle, including decreased blood flow and increased blood pressure [9,11,29]. These physiologic modifications can result in localized gangrene and the loss of animal productivity [2,10].

The combined incubation with 5-HT and ERV did not potentiate the inhibitory effect on contractility compared with these substances individually. The competition for receptors may have reduced the potential inhibitory activity when they were combined. These results indicate that an increase in 5-HT has the potential to mitigate vasoconstriction caused by ergot alkaloids and the refinement of optimal concentrations is warranted. The reduction of the contractile response of vascular smooth muscle to norepinephrine after prolonged exposure to 5-HT or ERV indicates that the residual effects of ergot alkaloids can be associated with triggering the G-protein coupled pathways that can be activated by serotonergic as well as adrenergic receptors. By simultaneously administering ERV and a selective agonist for the 5-HT_2A_ receptor, Trotta et al. [5] observed that the increase in the agonist concentration decreased the contraction caused by ERV in ruminal and mesenteric veins. The use of pharmacologically similar agents as a substitute for an undesired drug is considered agonist therapy [30]. Its efficacy has been proven for dependence on many substances, such as nicotine and opioids [31,32]. The kinetics of ergovaline at vascular serotonergic receptors complicates the development of treatment protocols, yet the prophylactic administration of molecules to modify the 5-HT receptors binding might be effective [33].

In contrast with many illicit drugs, ergotism acts directly on monoamine receptors instead of causing an increase of monoamines in the presynaptic terminal [2]. Thus, strategies to avoid receptor binding potentially achieve better results by reducing neuronal overstimulation and prolonged signaling. Ergot alkaloids (e.g., ergotamine) are extensively metabolized by the liver and cleared from the blood by first-pass hepatic metabolism [34,35]. Jaussaud [24] observed a half-life of 23.6 min for ERV in sheep, which suggests that strategies to avoid receptor binding can accelerate its clearance, reducing the neuronal excitation by the circulating ergot alkaloid.

The 5-HT receptors have complex and integrated functions throughout the body. Although many 5-HT receptors can cause vasoconstriction, activation of the 5HT_7_ receptor can provoke a hypotensive response [36]. The stimulation of the 5-HT_7_ receptor reduces the contraction induced by the 5-HT_2A_ receptor in the isolated abdominal vena cava [36]. It has been shown that the chronic administration of 5-HT can provoke a sustained fall in blood pressure in rats [37]. Therefore, an increase of 5-HT has the potential to activate receptors for relaxation responses, as well as to compete with ergot alkaloids reducing the persistent overstimulation of the receptor with contractile responses.

## 4. Conclusions

The increased exposure of blood vessels to 5-HT appears to compete with ergot alkaloids through receptors in the vascular smooth muscle, indicating that the manipulation of 5-HT has the potential to be used as a treatment of the vasoconstrictive effects induced by ergovaline. However, it is not clear how prolonged exposure to 5-HT and ergot alkaloids can reduce receptor sensitivity. This is further complicated by the fact that ergovaline has a 5000-fold higher potency than 5-HT. Future research should focus on the study of mechanisms related to the induction of blood vessel relaxation when under prolonged serotonergic stimulation, test doses of 5-HT or related compounds, and the evaluation of these in animal models.

## 5. Materials and Methods

No live animals were involved in the experiments that make up this study; thus, approval from the University of Kentucky Animal Care and Use Committee was not required.

### 5.1. Tissue Collection

The cranial branch of the lateral saphenous vein was collected from 14 predominantly Angus heifers (448 ± 21 kg) immediately after slaughter at the University of Kentucky Meats Laboratory using procedures according to Klotz [38]. Veins from nine heifers were used for calculation of IC_50_ (12 vessels sections per treatment) and veins from five heifers were used for testing the IC_50_ doses (15 vessels sections per treatment). As part of a separate study, the heifers were kept in the barn and maintained on a silage-based diet for 17 days prior to slaughter. Prior to that, they were managed on endophyte-infected tall fescue pastures with the same silage diet for at least 90 days. Segments (10 cm in length) of lateral saphenous vein at the cranial branch were removed and placed in a modified Krebs–Henseleit buffer for transport (95% O_2_/5% CO_2_; pH = 7.4; 11.1 mM D-glucose; 1.2 mM MgSO4; 1.2 mM KH_2_PO_4_; 4.7 mM KCl; 118.1 mM NaCl; 3.4 mM CaCl_2_; 24.9 mM NaHCO_3_; Sigma Chemical Co., St. Louis, MO), and were kept on ice during transportation from the collection site to the laboratory.

Blood vessels were dissected and the surrounding adipose and connective tissues were removed. The clean vessel segments were sliced into 2 mm cross-sections using an adjustable acrylic tissue matrix (Braintree Scientific, Inc., Braintree, MA, USA). The vessel cross-sections were inspected under magnification (12.5×) for abnormalities (e.g., structural damage incurred during dissection and points of vascular branches), and abnormal sections were discarded and replaced with viable sections.

### 5.2. Previous Exposure of Vascular 5-HT Receptors

To simulate the previous exposure of 5-HT receptors in the blood vessels to the treatments, the vein cross-sections were incubated at 37 °C in 5% of CO_2_ for 24 h in a 12-well culture plate (1 cross section/well) with 5 mL of Krebs–Henseleit buffer containing either control, serotonin, tall fescue seed extract (source of ergovaline; ERV), or a combination of the treatments. The previous exposures were conducted in two separate assays. The first assay determined the IC_50_ for 5-HT and the ergovaline-containing extract. The second assay evaluated the effect of IC_50_ doses of 5-HT and ergovaline determined in the previous assay, either individually or in combination.

### 5.3. IC_50_ Determination

To determine the IC_50_, stock solutions of 5-HT (Sigma-Aldrich, St. Louis, MO) were diluted to the corresponding final working concentrations of 1 × 10^−8^, 1 × 10^−7^, 1 × 10^−6^, 1 × 10^−5^ and 1 × 10^−4^ M. Working concentration ranges were prepared based on previous bovine vascular bioassay research using 5-HT [3]. Tall fescue seed extract was prepared and purified as described by Ji et al. [39] and Foote et al. [40]. Stock solutions of the extract were based on ERV concentrations (ergovaline + ergovalinine) and were prepared to final working concentrations of 1 × 10^−11^, 1 × 10^−10^, 1 × 10^−9^, 1 × 10^−8^ and 1 × 10^−7^ M. Working concentration ranges were prepared based on previous bovine vascular bioassay studies using ERV [3,41]. The vein segments were incubated with Krebs–Henseleit buffer alone or with working solutions of 5-HT or ERV for 24 h before contractile response evaluation.

### 5.4. Associated 5-HT and ERV Pre-Exposure

The blood vessels were incubated for 24 h with: (1) exclusively Krebs–Henseleit buffer; (2) Krebs–Henseleit buffer with 5-HT at IC_50_ concentration; (3) Krebs–Henseleit buffer with ERV at IC_50_ concentration; (4) Krebs–Henseleit buffer with 5-HT and ERV both at their respective IC_50_ concentrations. Following the completion of the 24 h pre-incubation period, the contractile response to increasing concentrations of 5-HT of each vein cross-section was evaluated.

### 5.5. Vascular Dimensions

Following the 24 h incubation, cross-sections were removed from treatment-containing buffer and examined using a dissecting microscope (Stemi 2000-C, Carl Zeiss Inc., Oberkochen, Germany) at 12.5× magnification to measure dimensions of the vessels. Vessel cross-sections were then transferred to a treatment-free Krebs–Henseleit buffer until mounting in the myograph chambers.

### 5.6. Contractile Response

After the 24 h incubation period of the treatments, blood vessels were mounted onto luminal supports in individual chambers of a multimyograph (DMT 610M, Danish Myo Technology, Atlanta, GA) with 5 mL Krebs–Henseleit buffer and constant gassing (95% O_2_/5% CO_2_; pH = 7.4; 37 °C). For each myograph experiment, the incubation treatments were run in duplicate during the determination of 5-HT and ERV IC_50_ and run in triplicate during evaluation of the determined IC_50_ doses associated with 5-HT and ERV.

The incubation buffer had the same composition of the transport buffer plus 3 × 10^–5^ M desipramine (Sigma Chemical, Co.) and 1 × 10^–6^ M propranolol (Sigma Chemical, Co). These compounds were included to inhibit biogenic amine reuptake and non-specific binding to β-adrenergic receptors, respectively. After mounting the blood vessels on the luminal supports in the myograph, an equilibration period was conducted with constant gassing (95% O_2_/5% CO_2_; pH = 7.4; 37 °C) for 90 min, with buffer changes every 15 min to allow blood vessels to reach a resting tension of approximately 1 g. At completion of the 90 min equilibration period, 1 × 10^−4^ M norepinephrine (Sigma Chemical, Co) was added to each chamber and incubated for 15 min. This served as a reference and to confirm vessel responsiveness and viability. Myograph chambers were then emptied and refilled with incubation buffer to remove norepinephrine and allow the vessels to return to an approximate 1 g tension. Once vessels returned to a resting tension, cumulative additions of 5-HT were added to reach the final concentrations of 5 × 10^−8^, 1 × 10^−7^, 5 × 10^−7^, 1 × 10^−6^, 5 × 10^−6^, 1 × 10^−5^, 5 × 10^−5^, and 1 × 10^−4^ M to build the contractile response curve. Afterwards, 1 × 10^−4^ M norepinephrine was again added to each chamber for evaluation of the final contractile response. Each 5-HT addition occurred in 15 min intervals in order of increasing concentration. Each cycle (addition) was a 9 min treatment incubation period, two 2.5 min buffer washes, a third buffer replacement, and followed by a 1 min recovery period leading into the next 5-HT addition.

### 5.7. Data Analysis

Isometric contractions in blood vessels to norepinephrine and 5-HT were digitized and recorded in grams of tension using PowerLab 16/35 and Chart software (version 8, ADInstruments, Colorado Springs, CO, USA). Baseline tensions were recorded before addition of each compound. For all contractile response data, the maximum tension (measured in g) during the 9 min incubation was recorded as the contractile response and corrected for baseline tension.

Due to variation between animals and tissues, contractile response data were normalized as a percentage of the maximum of tension induced by the reference addition of norepinephrine to compensate for differences in vessel responsiveness. Vessel contractile response data were reported as the percentage mean contractile response ± SEM of the maximum contractile response produced by the 1 × 10^−4^ M norepinephrine reference addition in vessels incubated for 24 h without treatment compounds. All contractile response data presented were plotted using GraphPad Prism software (San Diego, CA, USA). Sigmoidal concentration response curves to each treatment were calculated and plotted using a nonlinear regression equation:

$$y=\frac{100}{1+10{\;}^{\left(b-X\right)*a}}$$

where y is the contractile response, X is the concentration of the test solution, a is the Hill slope, and b is the concentration corresponding to the response midway between bottom and top (IC_50_).

### 5.8. Statistical Analysis

All data were analyzed as a completely randomized design using the MIXED model of SAS with the fixed effect of previous exposure treatment and the random effect of the incubation run.

The area under curve (AUC) was calculated using a linear trapezoidal method with baseline set to zero. The AUC was transformed, setting the control value to 100. Comparison of AUC and dimension of vessels to control in previous exposure doses was conducted by Dunnett test.

The heifer from which the vessel was collected and the myograph run were included as random effects to compare associated 5-HT and ERV previous exposure. Pairwise comparisons were conducted using the LSD test. Statistical significance was considered at *p* ≤ 0.05 and tendency at 0.05 < *p* ≤ 0.10.

## Figures and Tables

**Table 1:** Dimensions of bovine lateral saphenous veins after a 24 h incubation with buffer containing 5-HT.

	5-HT, log [M]						SE	*p*-Value
	0	−8	−7	−6	−5	−4		
Length, mm	2.14	2.17	2.27	2.36 *	2.22	2.21	0.06	0.001
ID, mm ^1^	1.41	1.32	0.89 *	0.87 *	0.89 *	0.86 *	0.19	<0.001
OD, mm ^2^	2.97	2.78	2.55 *	2.33 *	2.47 *	2.47 *	0.41	<0.001
Wall, mm ^3^	0.78	0.73	0.83	0.73	0.79	0.8	0.13	0.026

* Indicates a difference relative to control (0 M) by Dunnett test, *p* < 0.05. ^1^ Internal diameter. ^2^ External diameter. ^3^ Wall thickness.

**Table 2:** Dimensions of bovine lateral saphenous veins after a 24 h incubation with buffer containing ergovaline.

	Ergovaline ^1^, log [M]						SE	*p*-Value
	0	−11	−10	−9	−8	−7		
Length, mm	2.32	2.34	2.27	2.3	2.52	2.21	0.07	0.019
ID, mm ^2^	1.06	0.99	0.91	0.84 *	0.65 *	0.75 *	0.13	<0.001
OD, mm ^3^	2.76	2.57	2.51 *	2.40 *	2.29 *	2.43 *	0.36	<0.001
Wall, mm ^4^	0.85	0.79	0.80	0.78	0.82	0.84	0.12	0.463

* Indicates a difference relative to control (0 M) by Dunnett test, *p* < 0.05. ^1^ Tall fescue seed purified extract. ^2^ Internal diameter. ^3^ External diameter. ^4^ Wall thickness.

**Table 3:** Area under curve (AUC) of contractile concentration–response curve to increasing concentrations of serotonin (5-HT) and vascular dimensions of bovine lateral saphenous veins pre-incubated with buffer containing 5-HT, ergovaline (ERV), or both 5-HT and ERV.

	Treatments ^1^					*p*-Value ^2^		
	CTRL	5-HT	ERV	5-HT + ERV	SE	5-HT	ERV	5-HT × ERV
Length, mm	2.50	2.59	2.43	2.64	0.06	0.006	0.081	0.267
ID, mm ^5^	1.21	0.90	1.19	0.79	0.14	<0.001	0.44	0.571
OD, mm ^6^	3.16	2.84	3.12	2.76	0.17	<0.001	0.498	0.838
Wall, mm ^7^	0.976	0.969	0.964	0.986	0.05	0.785	0.924	0.565
AUC ^3^	98.4	37.9	72.7	40.7	9	<0.001	0.096	0.039
NE Response ^4^	98.9	49.8	92.3	51.7	6.8	<0.001	0.57	0.313

^1^ CTRL= only buffer; 5-HT = buffer with serotonin at IC_50_ concentration (7.57 × 10^−7^ M); ERV = buffer with ergovaline at IC_50_ concentration (1.57 × 10^−10^ M); 5-HT + ERV = buffer with serotonin (7.57 × 10^−7^ M) and ergovaline (1.57 × 10^−10^ M). ^2^ Main effects of serotonin (5-HT), ergovaline (ERV) and interaction (5-HT × ERV). ^3^ Area relative to control = 100. ^4^ Contractile response to norepinephrine (NE, 1 × 10^−4^ M). ^5^ Internal diameter. ^6^ External diameter. ^7^ Wall thickness.

## Data Availability

The data presented in this study are available on request from the corresponding author.
